# Chironomid midge sensitization in sewage workers: case study

**DOI:** 10.1111/j.1365-2915.2012.01058.x

**Published:** 2013-09

**Authors:** AI SELDÉN, A CALO, G MÖLLEBY, O HULTGREN

**Affiliations:** 1 Department of Occupational and Environmental Medicine, Örebro University HospitalÖrebro, Sweden; 2 Study Consultancy Inc., ÖrebroSweden; 3 Department of Clinical Microbiology, Örebro University Hospital, Örebro, Sweden and Department of Health and Medical Sciences, Örebro UniversityÖrebro, Sweden

**Keywords:** *Chironomus thummi*, fluorescence enzyme immunoassay, IgE, sensitization, sewage treatment, sewage workers

## Abstract

Non-biting chironomid midges (Diptera: Chironomidae) may cause sensitization and allergic reactions in humans and have recently been identified as a potential health problem in Swedish municipal sewage treatment plants. To investigate, on a pilot scale, the allergenic potential of chironomids in sewage workers, all workers (*n* = 8) at a sewage treatment plant and local controls (*n* = 16) completed a symptom questionnaire, underwent measurement of the fraction of nitric oxide in exhaled air, spirometry, and provided serum samples for the determination of atopy status and the prevalence of specific immunoglobulin E (IgE) antibodies against *Chironomus thummi* (*Chi t*) using a commercial fluorescence enzyme immunoassay (FEIA). Three sewage workers (38%) but no controls (0%) were FEIA positive for *C. thummi*-specific IgE antibodies (*P* < 0.05). No other health-related findings were significantly different between the groups. The study suggested that occupational exposure to Chironomids may cause sensitization with circulating IgE-antibodies in sewage workers.

Exposure to non-biting chironomid midges and their larvae (‘blood worms' and ‘red midge larvae’) can cause immunological sensitization and allergic symptoms in humans (Galindo *et al*., 1998). These phenomena have been reported in environmentally exposed populations worldwide (Cranston, 1988) but also in smaller groups of occupationally exposed subjects. For example, in a previous study involving 85 fish food workers and scientific laboratory personnel dealing with these insects, Baur & Liebers (1992) observed 24.7% of serum samples were positive for specific chironomid IgE antibodies. In contrast, Renström *et al*. (2011) found only two serum samples positive for chironomid IgE antibodies in 58 Swedish pet shop workers (3.4%).

Chironomids and spiders that feed on them are abundant in many Swedish municipal sewage treatment plants, where they are nuisances and may cause occupational health problems. In a recent survey of the managers at all Swedish municipal sewage plants, 58% of the respondents reported work environment problems associated with these insects (Lundström & Schäfer, 2006). To our knowledge, these issues have, however, not yet been addressed in studies on the occupational health of sewage workers (Thorn & Kerekes, 2001).

The present pilot study investigated the prevalence of chironomid sensitization in workers at a nearby municipal sewage treatment plant. For several years, swarms of chironomid midges have bred in the plant's indoor water pools, particularly during the summer and were regarded locally as occupational health hazards. Attempts to control the midge population using ultraviolet light-based insect traps failed, but generated an indoor organic dust derived from dead midges.

After appropriate ethical approval (Uppsala Ethical Review Board decision no. 2009/403), the entire workforce at the plant (*n* = 8) completed a symptom questionnaire in May to June 2010, as did a control group consisting of municipal workers who had not previously worked with sewage (*n* = 8) and hospital workers (*n* = 8). All groups underwent spirometry using a bellows instrument (Vitalograph S, Vitalograph, Buckingham, England) according to established procedures (Miller *et al*., 2005). Observed values for the vital capacity and the forced expiratory volume, 1 s, were compared with expected values derived from Hedenström *et al*. (1985) and Hedenström *et al*. (1986) for women and men, respectively. The fraction of nitric oxide in exhaled air (FE_NO_) was measured with a NIOX Mino (Aerocrine AB, Solna, Sweden). Subjects also provided blood serum samples, which were used to determine their atopy status (Phadiatop) and tested for specific IgE antibodies against *Chironomus thummi* (*Chi t)* using a fluorescence enzyme immunoassay (FEIA; ImmunoCAP 250®, Phadia AB, Uppsala, Sweden). Differences between the sewage workers and the merged control groups were investigated using Student's *t*-test and Fisher's exact test. As normality could not be assumed for FE_NO_, the Mann–Whitney *U*-test was used for this variable.

Three of the sewage workers (38%) and four of the controls (25%) were women. The sewage workers and controls were of a similar age (mean 53 years, range 27–62 and mean 55 years, range 27–63, respectively) and all subjects worked full-time (40 h/week). The groups did not differ significantly in terms of tobacco consumption or body mass index. A greater proportion of the sewage workers than the controls reported mucous membrane symptoms (affecting the eyes, nose and bronchi; Table 1), but neither these symptoms nor the spirometric findings, atopy in terms of a positive Phadiatop test or FE_NO_ levels differed significantly between the groups (Table 2). However, while three sewage workers (38%) were ImmunoCAP-positive for *Chi t*, none of the controls were (*P* = 0.03; Table 2). The ImmunoCAP-positive subjects had ratings of CAP-class 1–3, i.e. their serum samples contained between 0.35 and 17.4 kU/L of *Chi t*-specific IgE.

**Table 1:** Some questionnaire findings in Chironomid-exposed sewage workers and controls

	Sewage workers (*n* = 8)		Controls (*n* = 16)	
Symptom	*n*	*%*	*n*	*%*
Physician diagnosed asthma	2	25	0	0
Mucous membrane symptoms during the last week				
Itchy eyes	3	38	3	19
Runny nose	3	38	2	12
Sneezing	5	62*	3	19
Wheezy chest	2	25	0	0

* *P* = 0.06 (Fisher's exact test).

**Table 2:** Results of medical investigations and *Chironomus thummi*-specific IgE antibodies in sewage workers and controls

	Sewage workers (*n* = 8)		Controls (*n* = 16)	
Investigation	*n*	*%*	*n*	*%*
Lung function, % predicted; mean (range)				
Vital capacity	97 (84–114)		94 (73–116)	
Forced expiratory volume, 1 s	101 (90–118)		98 (72–123)	
Nitric oxide in exhaled air (FE_NO_), ppb; median (range)	19 (11–64)		18 (12–34)	
Phadiatop				
Positive	4	50	6	38
Negative	4	50	10	62
CAP-class (determined with FEIA*)				
0 (<0.35 kU/L)	5	62	16	100
1–3 (0.35–17.4 kU/L)	3	38†	0	0
4–6 (≥17.5 kU/L)	0	0	0	0

* Fluorescence enzyme immunoassay.

† *P* = 0.03 (Fisher's exact test).

Immunological sensitization and clinical allergy have previously been reported in aquarists using Chironomid larvae as a fish food, in fish food workers, in scientific workers exposed to these insects (Baur & Liebers, 1992) as well as in pet shop workers (Renström *et al*., 2011) but as far as we know, Chironomid-related health problems in sewage workers have not been previously examined. In the Nordic countries, indoor sewage water pools are common and provide fertile breeding grounds for insects such as chironomids and predatory spiders, creating a possibility for massive insect and allergen exposure. The abundance of insects is season dependent and comparatively low during the winter (Lundström & Schäfer, 2006) but the potential for exposure probably exists throughout the year.

The immunological findings of this study were rather straightforward, but their validity for the domestic sewage treatment industry in general is unclear. The results may have been influenced by selection and cluster effects and by cross-reactivity with allergens originating from, e.g. common mosquitoes, cockroaches, mites and shrimps **(**Galindo *et al*., 1998**)**. Interestingly, the high prevalence of chironomid sensitization (14–29%) previously observed in Swedish populations tested with *ad hoc* allergen extracts (Eriksson *et al*., 1989; van Kampen *et al*., 1994) was not seen in the current study (0% in unexposed controls) using a commercial extract, suggesting that the commercial preparation may be more specific.

The possible association between mucous membrane symptoms and the immunological findings was not further considered owing to the small size of the study. Moreover, no attempt was made to collect information on possible confounders in this respect, e.g. irritant gases such as ammonia or bacterial cell components such as endotoxin. Besides the presence of chironomids, however, ‘walk-through’ observations suggested a generally fair indoor air at the sewage treatment plant.

In conclusion, sewage workers are exposed to complex mixtures of chemical and biological agents, including insects and their larvae which may cause sensitization. These latter issues deserve further scrutiny.
